# Functional and Molecular Analysis of Human Osteoarthritic Chondrocytes Treated with Bone Marrow-Derived MSC-EVs

**DOI:** 10.3390/bioengineering11040388

**Published:** 2024-04-17

**Authors:** Annachiara Scalzone, Clara Sanjurjo-Rodríguez, Rolando Berlinguer-Palmini, Anne M. Dickinson, Elena Jones, Xiao-Nong Wang, Rachel E. Crossland

**Affiliations:** 1 Translational and Clinical Research Institute, Faculty of Medical Sciences, Newcastle University, Newcastle upon Tyne NE2 4HH, UK; 2 Centre for Advanced Biomaterials for Health Care@CRIB Istituto Italiano di Tecnologia, 80125 Napoli, Italy; 3 Leeds Institute of Rheumatic and Musculoskeletal Medicine, University of Leeds, Leeds LS7 4SA, UK; 4 Bioimaging Unit, Faculty of Medical Sciences, Newcastle University, Newcastle upon Tyne NE2 4HH, UK

**Keywords:** extracellular vesicles, mesenchymal stromal cell, chondrocyte, osteoarthritis, regenerative medicine

## Abstract

Osteoarthritis (OA) is a degenerative joint disease, causing impaired mobility. There are currently no effective therapies other than palliative treatment. Mesenchymal stromal cells (MSCs) and their secreted extracellular vesicles (MSC-EVs) have shown promise in attenuating OA progression, promoting chondral regeneration, and modulating joint inflammation. However, the precise molecular mechanism of action driving their beneficial effects has not been fully elucidated. In this study, we analyzed MSC-EV-treated human OA chondrocytes (OACs) to assess viability, proliferation, migration, cytokine and catabolic protein expression, and microRNA and mRNA profiles. We observed that MSC-EV-treated OACs displayed increased metabolic activity, proliferation, and migration compared to the controls. They produced decreased proinflammatory (Il-8 and IFN-γ) and increased anti-inflammatory (IL-13) cytokines, and lower levels of MMP13 protein coupled with reduced expression of MMP13 mRNA, as well as negative microRNA regulators of chondrogenesis (miR-145-5p and miR-21-5p). In 3D models, MSC-EV-treated OACs exhibited enhanced chondrogenesis-promoting features (elevated sGAG, ACAN, and aggrecan). MSC-EV treatment also reversed the pathological impact of IL-1β on chondrogenic gene expression and extracellular matrix component (ECM) production. Finally, MSC-EV-treated OACs demonstrated the enhanced expression of genes associated with cartilage function, collagen biosynthesis, and ECM organization and exhibited a signature of 24 differentially expressed microRNAs, associated with chondrogenesis-associated pathways and ECM interactions. In conclusion, our data provide new insights on the potential mechanism of action of MSC-EVs as a treatment option for early-stage OA, including transcriptomic analysis of MSC-EV-treated OA, which may pave the way for more targeted novel therapeutics.

## 1. Introduction

Osteoarthritis (OA) is a multifactorial joint disease associated with the progressive deterioration of cartilage and bone, resulting in joint failure. It is the most common form of arthritis, characterized by the degeneration of articular cartilage, with loss of matrix, fibrillation, formation of fissures, and ultimately apoptotic death of differentiated chondrocytes, leading to joint pain and functional limitation [1]. Key pathological changes in OA include severe inflammation, localized loss of articular cartilage, and remodeling of adjacent bone, with new bone formation at the joint margins [1]. Thus, OA may be regarded as a defective balance between the degradation and synthesis of joint tissues, whereby novel approaches with rebalancing potential may drive a therapeutic response.

Current treatments for OA are insufficient and usually palliative in nature, failing to prevent cartilage damage and subsequent destruction of other joint tissues. Mesenchymal stromal cells (MSCs) have shown promise in clinical trials as a regenerative therapy for OA by reducing synovitis, osteophyte formation, and cartilage degeneration [2]. However, their engraftment alone appears to be insufficient to account for the observed effects, especially when systemically infused [3]. Growing evidence suggests that the benefit of MSCs is trophic, via the secretion of paracrine factors including extracellular vesicles (EVs), which can activate and support endogenous cells in the damaged tissue [4]. MSC-EVs carry bioactive signaling molecules derived from their parent cell, such as proteins and small non-coding RNAs, which can interact with a variety of target cell types and modify their biological behaviors [5]. In recent years, there has been growing interest in the use of MSC-EVs as a potential therapeutic approach for treating OA. Pre-clinical studies have observed that MSC-EVs are able to recapitulate the therapeutic benefits of MSCs by attenuating OA progression, promoting chondral regeneration, and mediating cartilage repair via enhancing proliferation, attenuating apoptosis, stimulating extracellular matrix (ECM) production, and modulating joint inflammation [6,7,8]. Furthermore, molecularly modified MSC-EVs with elevated expression of selected miRNAs can promote cartilage regeneration and prevent OA [9,10,11]. Thus, MSC-EVs have shown the potential to serve as a novel cell-free therapy with significant advantages over their parent cells such as minimal risk of immunogenicity, non-self-replicating properties, ease of manufacturing, storage, and clinical administration [12]. They also have capacity for incorporation into state-of-the-art therapeutics such as scaffolds and hydrogels [13,14]. However, most studies to assess the effect of MSC-EVs on cartilage regeneration have been carried out in animal models [6,8,14,15,16], with few studies focusing on therapeutic MSC-EVs using in vitro human osteoarthritic chondrocyte models [7,17]. Furthermore, the underlying mechanisms driving MSC-EV therapeutic effects require further elucidation, and the molecular changes occurring in treated chondrocytes need to be fully dissected.

In this study, we sought to further investigate the therapeutic potential of MSC-EVs as an early treatment for OA by analyzing the functional and molecular characteristics of MSC-EV-treated human osteoarthritic chondrocytes (OACs) in comparison to untreated cells. To gain a greater understanding of the role of MSC-EVs on the coordinated therapeutic response to OA-associated damage, we examined the effects of MSC-EVs on OACs, by assessing their ability to promote cartilage repair and attenuate the degenerative processes associated with OA. Specifically, OACs were cultured in the absence or presence of MSC-EVs then subjected to cellular, molecular, metabolic, and functional assessment relevant to MSC-EV-mediated cartilage repair. The potential therapeutic effect of MSC-EVs was also assessed under interleukin 1ß-induced inflammatory conditions. We also expanded on the existing literature, by performing full microRNA and mRNA profiling of MSC-EV-treated OACs.

## 2. Materials and Methods

### 2.1. MSC Generation and Characterization

MSC samples were derived from different healthy donor bone marrow (BM) aspirates (surplus to hematopoietic stem cell transplant) with informed consent and Local Research Ethical Committee approval (NRES Committee North East—Newcastle & North Tyneside 2, 14/NE/1136). MSCs were isolated from BM mononuclear cells using a standard plastic adherence method, as previously described [18]. Briefly, MSCs were cultured in complete medium containing Dulbecco’s modified eagle medium (DMEM), 100 IU/mL penicillin, 100 μg/mL streptomycin, and 2 mM L-glutamine (Sigma-Aldrich, Burlington, MA, USA), supplemented with 5% human platelet lysate (PLTMax, MERCK, Darmstadt, Germany) and 2 IU/mL heparin. In vitro expanded MSCs were characterized at passage 3 as previously described [18] according to the criteria proposed by the International Society for Cell & Gene Therapy (ISCT) [19]. Passage 3 MSCs were used for EV collections.

### 2.2. MSC-EV Isolation

All EV samples were collected from individual MSC-conditioned medium by differential ultracentrifugation (UC), as previously described [18,20]. Briefly, 3 × 10^5^ MSCs were seeded in 75 cm^2^ flasks in 15 mL of complete medium. When the culture reached approximately 60% confluence, the complete medium was replaced with EV-depleted medium and cultured for a further 48 h prior to harvesting. MSC-conditioned medium was subjected to routine centrifugations at 400× *g* for 5 min (min) then 2000× *g* for 20 min to discard detached cells and debris before being transferred into UC tubes (Beckman Coulter) for sequential UC (10,000× *g* for 45 min then 100,000× *g* for 90 min in an Opti-XE-90 ultracentrifuge and 45Ti rotor (Beckman Coulter)). All centrifugations were performed at 4 °C. Following a wash with PBS (100,000× *g* for 90 min), the EV pellet was resuspended in 200–300 μL cold PBS then stored at −80 °C in aliquots.

### 2.3. MSC-EV Characterization

MSC-EVs were characterized using transmission electron microscopy (TEM), nanoparticle tracking analysis (NTA), flow cytometry (FC), and Western blot to demonstrate their morphology, particle size, and transmembrane protein expression, respectively.

TEM was performed using carbon-coated and plasma-etched 300-mesh grids (Gilder Grids, Grantham, UK) filmed with Pioloform^®^ (SPI Supplies, West Chester, PA, USA). EVs were resuspended in 100 μL PBS and a 10 µL droplet picked up by each grid, incubated for 5s, stained with uranyl acetate (Agar Scientific, Stansted, UK), and air-dried. Grids were examined using a Hitachi HT7800 TEM and digital images were collected using an Emsis Xarosa camera with Radius software v2.1 (Emsis, Munster, Germany), in conjunction with the Electron Microscopy Research Services, Newcastle University.

For FC assessment of the EV protein surface markers CD63, CD9, and CD81, EVs were coated onto 4 µm aldehyde/sulphate latex beads (ThermoFisher, Waltham, MA, USA), blocked with 1M Glycine (Sigma-Aldrich, Burlington, MA, USA), washed, and then incubated with anti-human PE CD63 (H5C6), PerCPCy5.5 CD9 (M-L13), and APC CD81 (JS-81) antibodies or corresponding isotype controls (all from BD Biosciences). Data acquisition was performed using an FACS Canto II cytometer (BD Biosciences, Franklin Lakes, NJ, USA) and analyzed with FlowJo v10.0 software (Tree Star Inc., Ashland, OR, USA).

NTA was performed using a NanoSight LM10-HS microscope (Malvern Panalytical Ltd., Malvern, UK) and NTA software v2.3 (Malvern Panalytical Ltd., Malvern, UK). Three 60 s recordings were recorded for each sample, diluted at 1:10,000 in sterile filtered PBS (Sigma). Only measurements with >1000 completed tracks were analyzed.

Western blots for Alix and Flotillin-1 were performed by lysis of EVs (2% sodium dodecyl sulfate (SDS)) and manual shearing. Protein quantification was determined using the Micro BCA™ Protein Assay Kit (ThermoFisher). Protein lysates were diluted and heated at 95° C, prior to loading onto a 4–20% Mini-PROTEAN^®^ TGX™ Precast Gel (Bio-Rad Laboratories, Hercules, CA, USA) alongside controls and molecular Precision Plus Protein™ Dual Colour Standards (Bio-Rad Laboratories, Hercules, CA, USA). Blots were incubated with 1:1000 primary antibody followed by 1:1500 secondary antibodies (Polyclonal Goat anti-Mouse, Daco, Glostrup, Denmark) before visualization under chemiluminescence detection using clarity reagent (Bio-Rad Laboratories, Hercules, CA, USA), the LI-COR Odyssey FC Imaging System, and Image Studio software v5.5.4 (LI-COR, Lincoln, NE, USA).

### 2.4. Chondrocyte Isolation and Culture

Human OACs were derived from articular cartilage samples obtained from OA patients undergoing total knee joint arthroplasty, with informed consent and Local Research Ethical Committee approval (REC reference: 19/LO/0389). Chondrocyte isolation and culture were performed as described previously [21]. Briefly, finely sliced articular cartilage pieces were washed in PBS then subjected to sequential enzymatic digestion in hyaluronidase (1 mg/mL in PBS) for 15 min, trypsin (2.5 mg/mL in PBS) for 30 min, and Type 1 collagenase overnight (2 mg/mL in serum-free DMEM supplemented with 2 mM glutamine, 100 IU/mL penicillin, 100 μg/mL streptomycin, and 2.5 μg/mL amphotericin B) (all Sigma-Aldrich). The same medium (+10% FCS) was used to resuspend the resulting cells, which were seeded at 2 × 10^6^ cells per 75 cm^2^ flask in 15 mL medium. Passage 1 cells were used in downstream experiments with MSC-EVs.

### 2.5. MSC-EV Uptake by Chondrocytes

To assess uptake of MSC-EVs into OACs, EVs were labeled with PKH26 Red Fluorescent Cell Linker Kit (Sigma), following the manufacturer’s protocol. OACs were plated on Poly-L-Lysine-coated coverslips at 1 × 10^4^ cells/100 μL and incubated with labeled MSC-EVs for 1 h at 37 °C followed by fixation with 2% paraformaldehyde for 15 min and permeabilization with 0.1% TritonX-100 for 5 min. The cells were incubated with Wheat Germ Agglutinin Alexa Fluor^®^ 488 conjugate (10 μg/mL, Invitrogen, Waltham, MA, USA) for 10 min in the dark to stain the cell membrane, then mounted using Vectashield medium with DAPI for nuclei staining. The cells were imaged using a Leica SP8 confocal 3D Super resolution microscope.

### 2.6. Viability Assay

OAC viability was assessed using CellTiter-Glo 2.0 assay following the manufacturer’s protocol (Promega, Madison, WI, USA). Briefly, MSC-EVs from 5 × 10^4^ MSCs were co-cultured with 5 × 10^3^ OACs in 100 µL of EV-depleted DMEM:F12/10%FBS in opaque 96-well plates (Nunclon delta, Waltham, MA, USA) [22]. All samples were plated as triplicate wells and OACs cultured with EV-depleted medium only as controls. Two doses of MSC-EVs were administrated on day 0 and day 3. An amount of 100 µL of equilibrated CellTiter-Glo 2.0 reagent was added on day 5 and luminescence was measured using the Spark Multimode Microplate Reader (Tecan, Mannedorf, Switzerland) and the Sparkcontrol method editor software v3.2 (Tecan, Mannedorf, Switzerland). This assessment included 3 OAC samples, each was treated with EVs from 3 different MSCs.

### 2.7. Scratch and Migration Assays

For scratch assays, OACs were cultured in 24-well tissue culture dishes to 80% confluency. A scratch of ≈0.4–0.5 mm was made using a sterile 10 µL pipette tip. Each well was washed three times with particle-free PBS to remove residual cells prior to addition of serum-free medium for control, and SFM + MSC-EV (1:4 ratio OAC:MSC-EV) for test conditions. The plate was re-incubated on a Nikon BioStation CT under SCC to monitor wound closure by imaging at 20 min intervals for 24 h. The scratch area was measured using the BioStation IM/IM-Q Ver2.23 software.

For migration assays, transwell filters (0.8 µm pore size) (Corning, New York, NY, USA) were coated with fibronectin (1 mg/µL) (Sigma) prepared to a 10 µg/cm^2^ coating density, then blocked with 0.1% FBS. OACs were added to the transwell at 2 × 10^4^ seeding density in 24-well plates containing DMEM, 0.1% FBS, and appropriate test conditions (negative control (0.1% FBS), positive control (10% FBS) or MSC-EV-treated (1:4 ratio OAC:MSC-EV)) and cultured for 24 h. Transwell filters were swabbed and methanol fixed (Sigma). Cells were stained using the Diff-Quick kit (Fisher Scientific, Waltham, MA, USA) followed by ethanol dehydration (50%, 70%, 100%) and migrated cells were visualized using the EVOS XL Core cell imaging system (Life Technologies, Carlsbad, CA, USA).

### 2.8. Secreted Protein, Cytokine, and Chemokine Assessment

Expression of IFN-γ, IL-8, IL-13 was assessed in chondrocyte-conditioned media supernatants by electrochemiluminescence immunoassay using the V-PLEX Proinflammatory Panel 1 Human Kit and Meso Quickplex Sq 120 instrument (Mesoscale Discovery, Rockville, MD, USA), based on 1:10 dilutions and following the manufacturer’s recommendations. The lower limits of detection (LLOD) ranged between 0.01 and 0.89 pg/mL. Secreted MMP-13 protein was assessed using commercially available ELISA kits (MMP-13: Human pro-MMP-13 Quantikine ELISA Kit, R&D Systems, Minneapolis, MN, USA) according to the manufacturer’s instructions at optimized dilutions (1:10). Absorbance was measured using a Microplate Reader (Thermo Labsystems Multiskan Ascent 354, Thermofisher, Waltham, MA, USA) at the manufacturer’s recommended wavelengths, and concentrations were obtained from standard curves.

### 2.9. Spheroid Culture of Chondrocytes

EVs isolated from 6 × 10^6^ MSCs were added to 6 × 10^5^ OACs and resuspended in DMEM:F12/10%FCS media. The cell suspension was plated in triplicates into cell-repellent U-bottom 96-well plates at 2 × 10^5^ OACs/well (Kremsmunster, Austria). The plate was centrifuged at 100× *g* for 5 min then cultured in a 5% CO2 incubator at 37 °C. Spheroids generated without MSC-EVs served as negative controls. Media were changed every 2 days. Cell pellets were processed for RNA isolation, sulphated glycosaminoglycans (sGAG) quantification, and immunohistochemistry (IHC) after 2 and 21 days. To examine whether MSC-EVs could diminish the inflammatory environment-induced OA phenotype, the spheroid cultures were also established in the presence or absence of IL-1β (1 ng/mL) for both MSC-EV-treated and untreated conditions. In this setting, the spheroids were processed for RNA isolation and sGAG at day 10 and 21, respectively [23].

### 2.10. Sulphated GAG Quantification

Spheroid samples were digested overnight at 65 °C in 100 µL of papain digestion buffer (1 mg/mL) (containing 2 mM Acetyl Cysteine and 2 mM EDTA in 50 mM sodium phosphate) (Sigma). The supernatant was collected by centrifugation for sGAG quantification, using the Blyscan Assay kit (Biocolor, Carrickfergus, Co Antrim, UK), according to the manufacturer’s protocol. sGAG levels in the samples were calculated from a linear standard curve of chondroitin-4-sulfate, range 0–5 µg of sGAGs.

### 2.11. Histology and Immunohistochemistry

Histological and immunohistochemical (IHC) analysis was performed on OAC pellet sections at day 21, following previously established protocols. Briefly, samples were fixed (10% formalin), transferred to 70% Ethanol (EtOH), then to histology cassettes. Pellets were paraffin-embedded following routine histological procedures and sectioned in 7 µm thick slices. The slices were fixed on glass slides, deparaffined, and then stained, according to standard procedures, with Haematoxylin and Eosin for cell nuclei and cytoplasm and with toluidine blue for GAGs [24].

For IHC, fixed and washed tissue slices were permeabilized with Triton 100x solution (0.1% *w*/*v* in PBS), then immersed in BSA solution (2% *w*/*v* in PBS) and incubated with primary antibody (mouse anti-aggrecan, ab3778 Abcam) (1:50 in BSA solution) for 2 h at room temperature. After washing with PBS, secondary antibody (Goat anti-Mouse IgG (H&L)—Alexa Fluor™ 488, A-11001, Thermo Fisher Scientific) was added to the slices for 1 h (1:1000 in BSA solution). Nuclei were stained with DAPI mounting medium (Thermo Fisher Scientific), according to the manufacturer’s instructions, and a coverslip applied. Slides were imaged using an EVOS M5000 168 microscope with fluorescence at 20× and 40× magnification.

### 2.12. RNA Isolation and Quantification

Total RNA was isolated from EVs using the Total Exosome and Protein Isolation Kit (Invitrogen, Waltham, MA, USA) and from cells using the miRNeasy Mini Kit (Qiagen, Hilden, Germany), as per the manufacturer’s instructions. For NanoString profiling, RNA was concentrated to 25 μL using Amicon Ultra-0.5 Centrifugal Filter Units (Merck Millipore, Darmstadt, Germany). All RNA was quantified using the Bioanalyzer and RNA 6000 Pico kit (Agilent, Santa Clara, CA, USA), RNA 6000 Nano kit (Agilent, Santa Clara, CA, USA), or the NanoDrop 1000 spectrophotometer (ThermoFisher, Waltham, MA, USA), as appropriate.

### 2.13. Quantitative Real-Time PCR

MicroRNA and endogenous control-specific cDNA (HY3 and U6) was generated using TaqMan^®^ MicroRNA Assays or TaqMan^®^ Control Assays and the TaqMan^®^ MicroRNA Reverse Transcription kit (ThermoFisher), according to the supplier’s protocol. Each reaction incorporated TaqMan^®^ MicroRNA Assays or TaqMan^®^ Control Assays (ThermoFisher) and SensiFast Probe Hi-Rox reagent (Bioline) (Supplementary Table S1). Gene expression was performed by reverse transcription using random hexamer primers and the High-Capacity cDNA Reverse Transcription kit (ThermoFisher), followed by gene-specific amplification using TaqMan Gene Expression Assays (ThermoFisher) (Supplementary Table S1) and SensiFast Probe Hi-Rox reagent, according to the supplier’s protocols. Thermal cycling was performed in triplicate using the 7900HT Real-Time PCR System (ThermoFisher).

### 2.14. NanoString

Total RNA was profiled using the nCounter^®^ Human v4.0 miRNA Expression Assay Kit (NanoString Technologies, Seattle, WA, USA) as previously described [25], incorporating *n* = 799 mature microRNAs and positive, negative, ligation, and housekeeping controls. Data normalization was performed using the NanoStringDiff normalization procedure. Normalized counts were filtered to exclude those with <5 counts in <50% of samples. Batch effects were estimated using surrogate variable analysis and were removed using the removeBatchEffect function within the limma package for visualization; batch terms were added into the model for differential expression analysis.

### 2.15. RNA-Seq

Total RNA samples (n = 24) were prepared using the ‘TruSeq Stranded mRNA’ kit (Illumina) following the manufacturer’s instructions, pooled into one library, and sequenced on a NextSeq 500 ‘High-Output—75 cycle’ (Illumina, San Diego, CA, USA) sequencing run; equivalent to >15 million 75 bp single reads per sample. Sequencing services were provided by the Genomics Core Facility, Newcastle University. The FastQC tool was used to check the quality of raw sequencing reads; all samples passed quality checks. Each sample was mapped to the hg38 reference genome using Salmon [26], achieving a ~90% mapping rate, and differential testing was performed using DESeq2 [27], incorporating SVA [28] to estimate batch as well as OAC donor in the model.

### 2.16. Pathway and Gene Enrichment Analysis

Target KEGG pathways for selected microRNAs were predicted using miRPath v.3 (Diana Tools), based on microT-CDS predicted targets and TarBase v.8 experimentally supported targets, incorporating genes’ union with *p* < 0.05, microT threshold = 0.8, and FDR correction [29]. Gene canonical pathways, upstream regulators, diseases and functions, and mechanistic networks were performed using Ingenuity Pathway Analysis (IPA) (Qiagen, Hilden, Germany). MicroRNA–mRNA pairings were analyzed using the IPA MicroRNA Target Filter, based on experimentally validated interactions from TarBase and miRecords, and predicted interactions from TargetScan. To link RNA-Seq gene expression changes to functional data, fold changes were correlated against skeletal transcriptomic datasets using SkeletalVis to identify the most similar and dissimilar datasets (http://skeletalvis.ncl.ac.uk/skeletal [30] (accessed on 6 August 2021)). Both correlation and overlap of genes > 1.5 absolute fold changes were considered, and overlap scores were normalized using z-scores > 2 used to identify the most similar/dissimilar datasets based on a background of chondrocyte-related datasets. Predicted microRNA targets were identified using miRWalk [31], based on TarPmiR, TargetScan, miRDB, and miRTarBase.

### 2.17. Statistical Analysis

TaqMan microRNA expression was analyzed as relative expression to the mean of endogenous controls using the 2-(ΔCt) method, in the absence of an appropriate calibrator. The expression of genes of interest at day 21 was normalized to GAPDH and presented as relative expression using the 2-(ΔΔCt) method, using the expression levels of the day 2 control conditions as calibrator. All datasets were tested for normal distribution using the D’Agnostino and Pearson test, or Shapiro–Wilk test for smaller datasets. For viability assays, data were analyzed using the Wilcoxon matched pairs signed-rank test. For migration assay, sGAG quantification, and gene expression, data were analyzed using the paired *t*-test or one-way ANOVA, as appropriate. For scratch assay, data were analyzed using a mixed-effects model, with Sidak’s multiple comparisons test.

## 3. Results

### 3.1. Characteristics of MSCs and MSC-EVs

**Figure 1:**
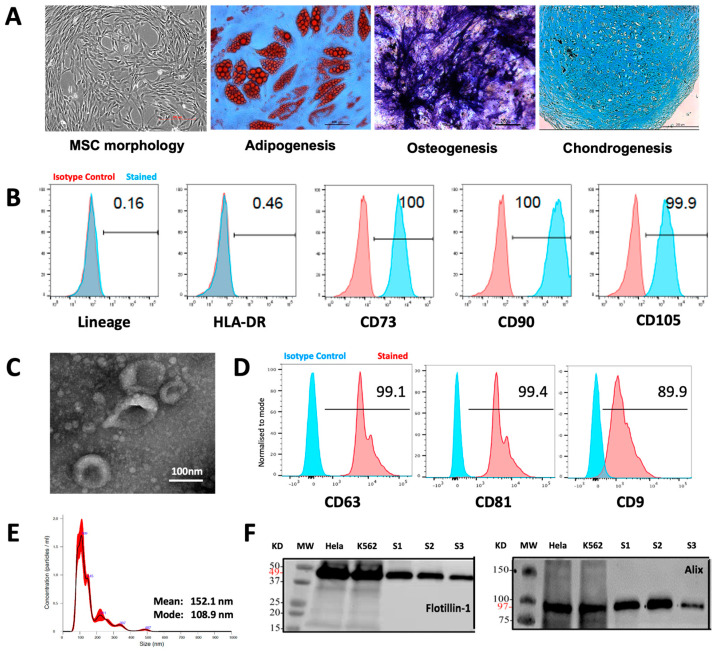
Characteristics of MSCs and MSC-EVs. (**A**) MSC morphology and tri-lineage potential (adipogenesis, osteogenesis, chondrogenesis) assessment. (**B**) FC histogram showing MSC surface phenotype profiles for lineage specific markers (CD14, CD19, CD34, CD45), HLA-DR, CD105, CD73, and CD90. (**C**) TEM image showing EV morphology. (**D** FC histogram showing positive expression of EV markers CD63, CD81, and CD9. (**E**) NTA analysis showing EV size distribution. (**F**) Western blot analysis showing positive expression of EV markers Flotillin-1 and Alix in three independent MSC-EV examples (S1 to S3).

MSCs were generated from healthy donor BM aspirates using the plastic adhesion method and confirmed to comply with the International Society for Cell and Gene Therapy (ISCT) criteria [19], demonstrating characteristic morphology, trilineage potential for adipogenesis, osteogenesis, and chondrogenesis (Figure 1a), and a surface phenotype < 2% positive for lineage specific markers (CD14, CD19, CD34, CD45, and HLA-DR), while being > 95% positive for the expression of CD105, CD73, and CD90 (Figure 1b). MSC-EVs were isolated from MSC-conditioned media using differential UC and characterized according to the International Society for Extracellular Vesicle (ISEV) guidelines [28], demonstrating typical cup-shaped vesicular morphology by TEM (Figure 1c), positivity for CD63, CD81, and CD9 markers by FC assessment (Figure 1d), a modal vesicle size of 108.9 nm, within the expected EV size range (30–200 nm) by NTA (Figure 1e), and positivity for Flotillin-1 and Alix, according to Western blot analysis (Figure 1f).

### 3.2. MSC-EV-Treated OACs Display Increased Viability, Proliferation, and Migration

**Figure 2:**
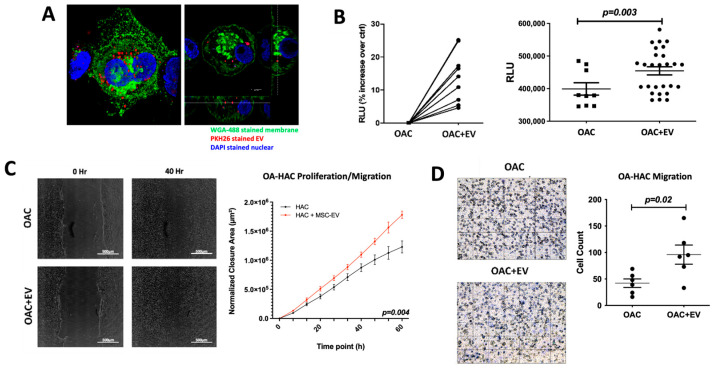
MSC-EV-treated OACs’ EV uptake, viability, mobility, and migration. (**A**) Confocal microscopy images to show MSC-EV-treated OACs. OACs internalize MSC-EVs after 1 h of co-incubation. Cut view confirms internalization of MSC-EVs. (**B**) Analysis of metabolic activity of OACs and EV-treated OACs (OAC + EV) via CellTiter-Glo 2.0 assay after 5 days of culture. Significance was calculated using the Wilcoxon matched pairs signed-rank test. (**C**) Two-dimensional scratch assay: example images for OAC and OAC + EV samples at 0 and 40 h post-scratch. Significance was calculated using a mixed-effects model, with Sidak’s multiple comparisons test. (**D**) Cell migration assay: example images are shown for OAC and OAC + EV samples, assessed using a transwell filter system. Total cell count is shown for OAC and OAC ± EV. Significance was calculated using the paired *t*-test. (**B**–**D**) Lines represent mean ± SEM. The significance threshold for *p*-values was *p* < 0.05.

Transferring EV cargo into target cells is a major mechanism for EV-mediated molecular and functional modulation of the target cells. To confirm uptake of MSC-EVs by OACs, OACs were co-cultured with PKH26-stained MSC-EVs. The presence of MSC-EVs inside OACs was observed after only one hour of co-incubation (Figure 2a), indicating rapid communication potential between MSC-EVs and OACs.

The ability of internalized MSC-EVs to influence OACs’ viability, proliferation, and migration was assessed in a monolayer co-culture model. An ATP-based viability assay revealed that MSC-EV-treated OACs (OAC + EV) displayed significantly higher ATP levels than untreated controls (*p* = 0.003) (Figure 2b). To assess the ability of MSC-EVs to enhance OAC motility and migration, which may be a contributing factor to cartilage repair, a 2D scratch assay and cell migration assay were performed. In the scratch assay, MSC-EV treatment significantly increased OAC proliferation/migration compared to untreated OACs (*p* = 0.004) (Figure 2c). Similarly, MSC-EV-treated OACs exhibited a significantly enhanced ability to migrate in a transwell migration assay (*p* = 0.02) (Figure 2d).

### 3.3. MSC-EV Switch OAC Cytokine, Catabolic Protein, Gene, and MicroRNA Expression to Favor Cartilage Repair

**Figure 3:**
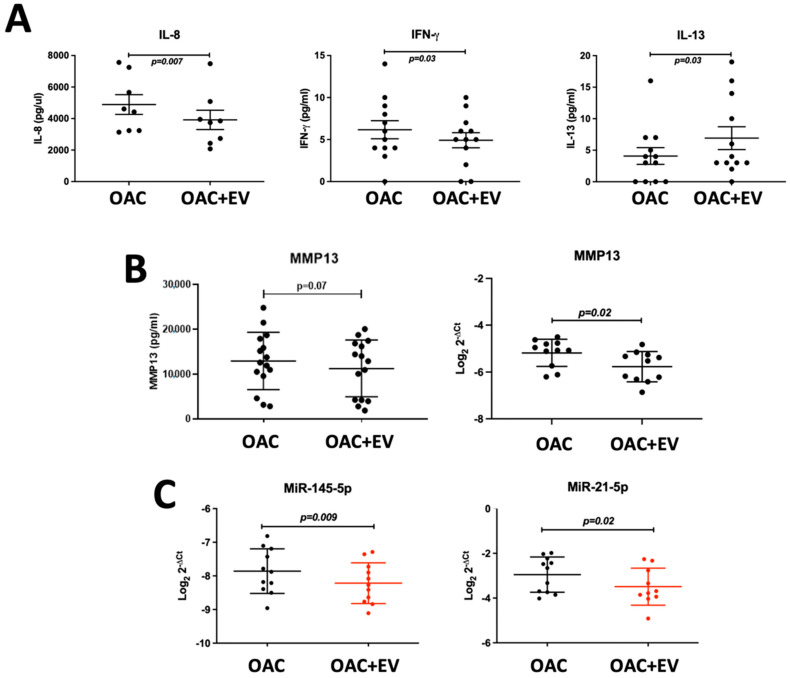
MSC-EV-treated OACs’ cytokine, catabolic protein, gene, and microRNA expression. (**A**) Secreted cytokine production (IFN-γ, IL-8, IL-13) by MSC-EV-treated OACs by multiplex immunoassay in n = 12 OAC and OAC + EV. (**B**) Secreted MMP13 protein production by ELISA in n = 15 OAC and OAC + EV, and gene expression assessed by qRT-PCR in 2D monolayer cultures in n = 11 matched OAC controls and OAC + EV. (**C**) MicroRNA expression assessed by qRT-PCR in 2D monolayer cultures in n = 11 matched OAC controls and OAC + EV. (**A**–**C**) Expression differences between groups were calculated using the paired *t*-test, and lines represent mean ± SEM. The significance threshold for *p*-values was *p* < 0.05.

To assess the effect of MSC-EV treatment on OAC cytokine and catabolic protein production, we analyzed the supernatants collected from 24 h monolayer co-cultures of OACs in the presence or absence of MSC-EVs using a multiplex immunoassay. MSC-EV-treated OACs produced significantly decreased pro-inflammatory cytokines IL-8 (*p* = 0.007) and IFN-γ (*p* = 0.03) and increased anti-inflammatory cytokine IL-13 production (*p* = 0.03) (Figure 3a). The production of other pro-inflammatory cytokines (IL-1β, IL-6 and TNF-α) was below the detectable level at the 24 h co-culture time point. Production of the catabolic ECM proteinase MMP13 was also decreased in MSC-EV-treated OACs (*p* = 0.07) (Figure 3b), as detected by the ELISA analysis of the co-culture supernatants. MMP13 downregulation (*p* = 0.02) after 24 h co-culture was confirmed at the mRNA level (Figure 3b).

Expression of microRNAs associated with the regulation of chondrogenesis was also assessed in the monolayer system. Known negative regulators of chondrogenesis were downregulated in EV-treated OACs compared to the controls (miR-145-5p *p* = 0.009, miR-21-5p *p* = 0.02) (Figure 3c).

### 3.4. MSC-EV-Treated OACs Display a Pattern of Gene Expression and sGAG Production in Favor of Chondrogenesis in a 3D Spheroid Model

**Figure 4:**
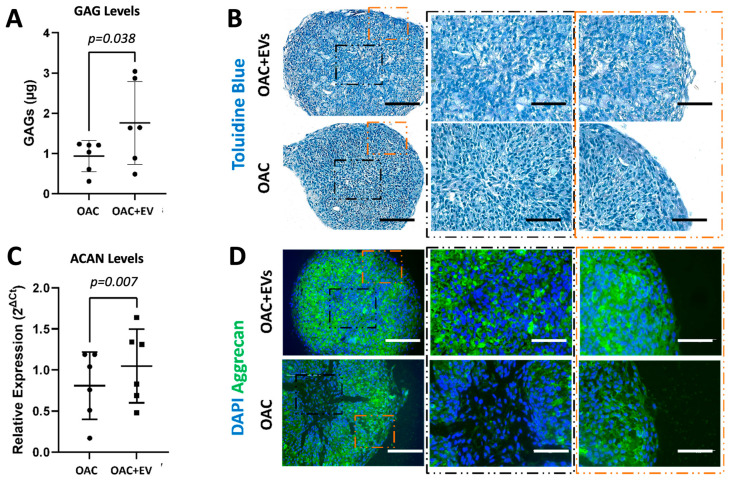
Chondrogenic potential of MSC-EV-treated OACs in a 3D spheroid model. (**A**) Quantification of sGAG production in OAC and OAC + EV samples. (**B**) Representative histological analysis of toluidine blue, which stains sGAGs, on tissue slices from three independent experiments (scale bar = 200 µm). Zoomed image is shown in the center and in the peripheral zone of the spheroid slice (scale bar = 125 µm). (**C**) ACAN relative expression via RT-qPCR, for OAC and OAC + EV, at day 21 with respect to day 2. (**D**) Representative IHC of aggrecan on tissue slices for OAC and OAC + EV samples (scale bar = 200 µm). Zoomed image is shown in the center and in the peripheral zone of the spheroid slice (scale bar = 125 µm). (**A**,**C**) Significance was calculated using the paired *t*-test and lines represent mean ± SEM. The significance threshold for *p*-values was *p* < 0.05.

To assess the effect of MSC-EVs on the expression of recognized chondrogenic and catabolic genes in more physiologically relevant models of OAC growth, 3D spheroid in vitro cultures were established. These comprised OACs co-cultured for 21 days in the absence or presence of MSC-EVs. OACs treated with MSC-EVs demonstrated significantly higher levels of sGAGs (1.8 ± 0.9 µg) compared to untreated cells (0.9 ± 0.4 µg) after 21 days of culture (*p* = 0.038) (Figure 4a). Histological assessment of toluidine blue (TB) demonstrated higher levels of GAG-stained ECM in the spheroids formed by OAC + EV (Figure 4b). In addition, OAC + EV showed significantly increased levels of ACAN gene expression (*p* = 0.007) (Figure 4c), as well as higher aggrecan protein expression detected by IHC, whereby aggrecan staining was more uniform and intense in EV-OACs, compared to untreated controls, suggesting a chondrogenic-promoting effect by MSC-EVs (Figure 4d). There was no significant effect on differential gene expression in the model system when comparing between one or two doses of MSC-EV treatment (*p* > 0.05).

### 3.5. MSC-EVs Reverse the Pathological Impact of IL-1β on OAC Chondrogenic Gene Expression and ECM Component Production

**Figure 5:**
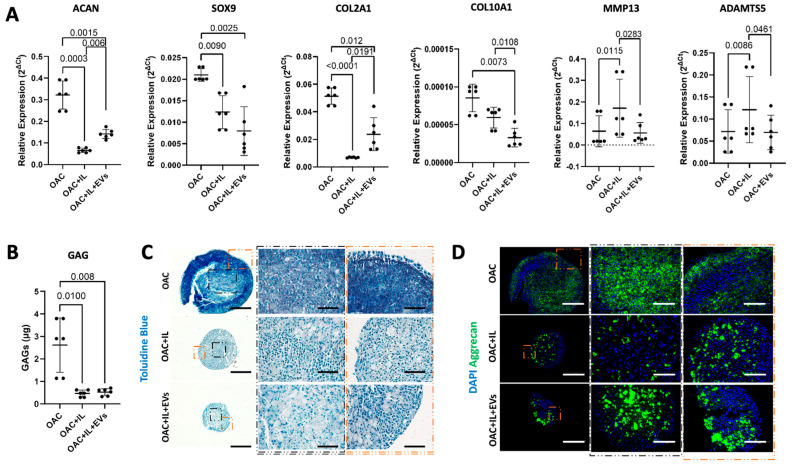
Effect of MSC-EVs and IL-1β on OAC chondrogenic gene expression and ECM component. (**A**) Relative gene expression of ACAN, SOX9, COL2A1, COL10A1, MMP13, and ADAMTS at day 10 with respect to day 2. (**B**) Quantification of sGAG production in digested spheroids formed from OAC, OAC + IL, and OAC + IL + EV samples. (**C**) Representative histological analysis of Toluidine blue staining GAGs from three independent experiments. Scale bars represent 200 µm and 125 µm, respectively, for original and zoomed-in areas, as indicated. (**D**) Representative IHC of aggrecan staining on tissue sections of OAC, OAC + IL, and OAC + IL + EV samples from three independent experiments. Scale bars represent 200 µm and 125 µm, respectively, for original and zoomed areas, as indicated. (**A**,**B**) Significance was calculated using one-way ANOVA and lines represent mean ± SEM. The significance threshold for *p*-values was *p* < 0.05.

We next developed the in vitro 3D OA model [23] to include the addition of the inflammatory cytokine IL-1β, to mimic the pathological features of OA. The capacity of MSC-EVs to reverse IL-1β-induced pathological expression patterns of chondrogenic and catabolic genes was then assessed. After 10 days of co-culture with MSC-EVs, the addition of IL-1β to the OAC model (OAC + IL) resulted in the downregulated expression of ACAN (*p* < 0.001), SOX-9 (*p* = 0.009), COL2A1 (*p* < 0.001), and COL10A1 (*p* < 0.01) and the upregulation of MMP13 (*p* = 0.012) and ADAMTS5 (*p* = 0.009) (Figure 5a). However, we observed that the addition of MSC-EVs during culture with IL-1β partially reversed this effect, leading to a significant increase in ACAN (*p* = 0.006) and COL2A1 (*p* = 0.019) expression, together with a decrease in MMP13 (*p* = 0.012) and ADAMTS5 (*p* = 0.046) (Figure 5a). A significant reversal of the effect of IL-1β on SOX9 by MSC-EVs was not observed. We observed that the OAC samples showed the most intense sGAG quantification (Figure 5b) and GAG staining (Figure 5c) compared to OAC + IL and OAC + IL + EV. In the presence of IL-1β, with and without EVs, the sample slices appeared fragmented and smaller, probably due to a decrease in OAC cell proliferation and the catabolic activity of aggrecanases. Aggrecan staining, while appearing more intense in the OAC samples, seemed to be more intense and even more homogenously distributed in the IL-1β-loaded samples in the presence of MSC-EVs (OAC + IL + EVs), compared to the OAC + IL (Figure 5d), confirming the trend of the gene expression results. Overall, the presence of MSC-EVs contributed to reversing the effect of IL-1β.

### 3.6. MSC-EVs Change the Global Gene Expression Profiles of Treated OACs

**Figure 6:**
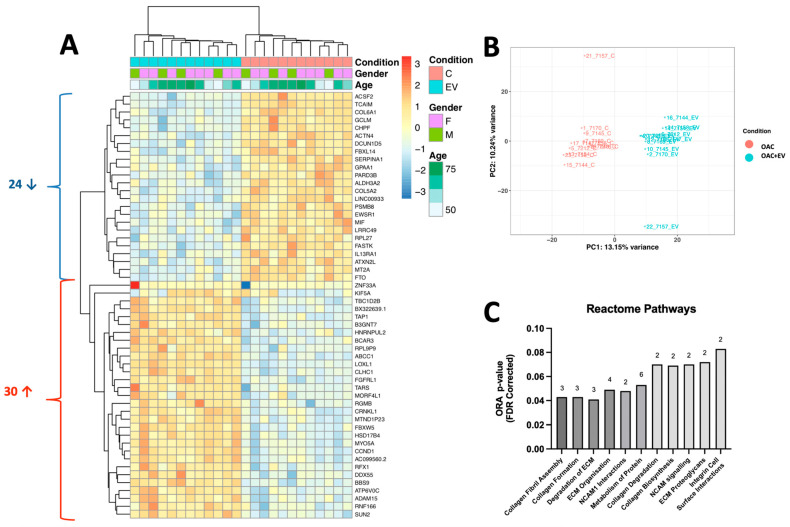
Gene expression profile of MSC-EV-treated OACs compared to OACs. (**A**) Heatmap shows unsupervised hierarchical clustering of 54 genes that were significantly differentially expressed between groups: each column represents an individual sample. OAC + EV are depicted in blue, while control OACs are depicted in pink. Patient gender (green, purple) and age (green scale) are also indicated. Relative expression changes are indicated by the color scale (red: high; blue: low). (**B**) Principal component analysis of MSC-EV-treated OACs vs. control OACs. (**C**) Reactome pathway analysis for genes significantly differentially expressed between OAC + EV and OAC controls. Both analyses indicate the number of genes implicated in each pathway.

To explore the broader impact of MSC-EV treatment on global gene expression in OACs, we performed RNA-Seq and NanoString microRNA profiling on OAC + EV (n = 3) in 2D monolayer co-cultures (n = 14). RNA-Seq analysis identified 54 genes that were significantly differentially expressed (DE) in OAC + EV compared to the untreated controls (Log2 FC Range = −9.51–19.60, *p* < 0.01). Among those, 24 genes were downregulated in OAC + EV and 30 genes were upregulated (Figure 6a). Principal component analysis demonstrated that the OAC + EV and OAC formed two distinct clusters (Figure 6b). The significantly differentially expressed genes between groups included those implicated in cartilage function (COL5A2, COL6A1, HSD17B4, ACTN4, CCND1, LOXL1), mapping to REACTOME pathways such as collagen biosynthesis, ECM organization, and interactions (Figure 6c).

To link the gene expression changes to functional data, fold changes identified in OAC + EV were correlated against a database of skeletal transcriptomic datasets, using SkeletalVis to identify the most similar and dissimilar datasets and assess the drivers of observed gene expression. SkeletalVis returned 27 skeletal transcriptomic datasets (Z-score range 2.01–5.29) from mouse and human models including synovium, embryo, cartilage, meniscus, and bone tissue with a total of 5 genes (B3GNT7, KIF5A, SERPINA1, TAP1, PSMB8) identified as upregulated in both the annotated datasets and our RNASeq data after MSC-EV treatment of OACs, while 2 genes (LRRC49, BBS9) were downregulated in both datasets (Table 1 and Figure 6a).

### 3.7. MSC-EVs Affect the MicroRNA Expression Profiles of Treated OACs

Molecular differences between the MSC-EV-treated OACs and controls were also explored at the microRNA level, by performing microRNA expression profiling (NanoString) in the same OAC + EV (n = 3) in 2D monolayer co-cultures (n = 14).

**Figure 7:**
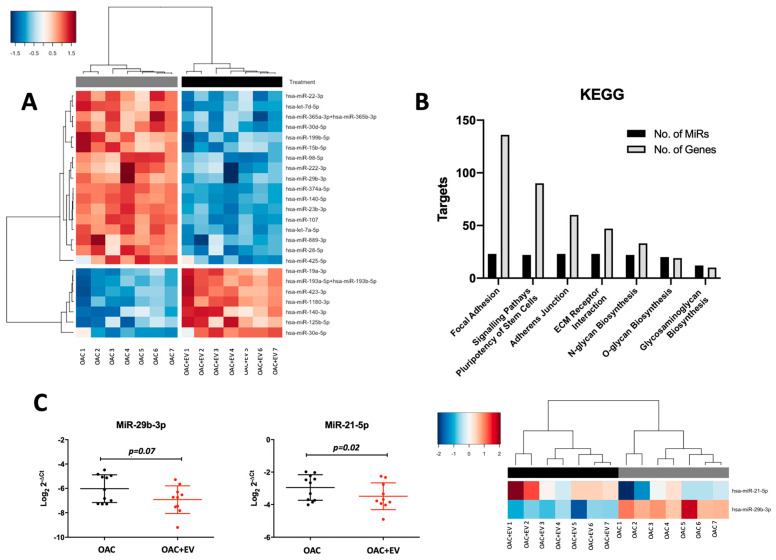
Differential microRNA expression profiles of OAC + EV and OAC. (**A**) NanoString microRNA expression profiling: heatmap shows unsupervised hierarchical clustering of significantly differentially expressed microRNAs (*p* < 0.05, n = 24), based on normalized expression counts, in OAC + EV and controls. Each column represents an individual sample. Relative expression changes are indicated by the color scale (red: high; blue: low). OAC + EV are depicted by black shading, while OAC are depicted by grey shading. (**B**) KEGG pathways analysis for the 24 differentially expressed microRNAs in OAC + EV vs. control OACs. Pathway analysis was performed using Diana Tools miRPath, based on TarBase v8.0 and the number of target genes and targeting microRNAs are plotted for each pathway (FDR-corrected *p*-values < 0.05). (**C**) Validation of selected microRNA expression in an independent cohort of OAC + EV and OAC controls (n = 11) by qRT-PCR. Lines represent mean ± SEM. Significance was calculated using the paired *t*-test, and the threshold for *p*-values was *p* < 0.05. Heatmap to show unsupervised hierarchical clustering of the same microRNAs according to NanoString data (n = 7 matched samples and controls).

Assessing the microRNA expression differences revealed 24 differentially expressed microRNAs (Log2 FC range = −1.30–1.22, *p*-value range *p* < 0.001–0.04), whereby 17 were downregulated (Log2 FC range = −1.30–−1.03, *p*-value range *p* < 0.001–0.04) and 7 were upregulated (Log2 FC range = 1.03–1.22, *p* < 0.001–0.04) in OAC + EV (Figure 7a).

The 24 differentially expressed microRNAs were associated with chondrogenesis-related KEGG pathways, including focal adhesion (136 genes, 23 microRNAs, *p* < 0.001), adherens junction (60 genes, 23 microRNAs, *p* < 0.001), ECM receptor interaction (47 genes, 23 microRNAs, *p* = 0.016), and glycosaminoglycan biosynthesis (10 genes, 12 microRNAs, *p* < 0.001) (Figure 7b).

The predicted targets of significantly differentially expressed microRNAs were identified using miRWalk and correlated with significantly differentially expressed genes identified by our RNASeq data. This analysis indicated that 34 of the genes differentially expressed in OAC + EV were predicted targets of microRNAs that were also significantly differentially expressed in OAC + EV. The Ingenuity Pathway Analysis (IPA) microRNA target filter showed targeting information for 22 of the 24 differentially expressed microRNA, of which 7 were experimentally observed to target 11 genes that were differentially expressed in OAC + EV by RNA-Seq with inverse pairing (Table 2). These included genes known to be implicated in chondrogenesis, ECM organization/interaction, and integrin interactions (COL5A1 targeted by miR-98-5p and miR-29b-3p, COL5A2 targeted by miR-30e-5p, SMAD3 targeted by miR-140-5p and miR-23a-3p, FGFR1 targeted by miR-16-5p, PLOD2 targeted by miR-30e-5p) (Table 2).

Selected differentially or highly expressed microRNAs were validated in an independent cohort of MSC-EV-treated OAC monolayer cultures (n = 11), confirming the findings of the NanoString profiling data (miR-29b-3p *p* = 0.07, miR-140-5p *p* = 0.03, miR-21-5p *p* = 0.02) (Figure 7c).

## 4. Discussion

This study describes the in-depth exploration of MSC-EVs and their mechanistic potential to enhance the efficacy of OA treatment. These small entities have demonstrated an exceptional ability to manifest anti-inflammatory and chondro-protective effects in preliminary studies using OA in vitro and in vivo animal models [32,33]. However, the mechanistic actions driving their beneficial effects, and the potential role of microRNAs in these processes, have yet to be fully elucidated. Progress in this area is almost certainly hindered by the lack of consistency between studies and models, with respect to multiple factors such as the source of cells, the type of EVs, the EV isolation method, EV dosage, co-culture conditions, stimulation, and lack of in vitro human models. Thus, in this study, we sought to expand the growing evidence for the therapeutic potential of MSC-derived EVs as an early treatment for OA, by conducting functional and molecular studies in clearly defined and well-accepted in vitro models [34], using EVs isolated from healthy-donor BM MSCs, characterized using internationally accepted standards [35]. Although adipose-derived or umbilical cord-derived MSCs can be used for MSC-EV production aimed at cartilage regeneration [36], BM MSC-EVs have been more commonly employed in pre-clinical and clinical investigations [37]. We have selected osteoarthritic chondrocytes (OACs) as target cells, in line with many previous studies [34], and because chondrocyte abnormalities present the earliest signs of OA, thus enabling targeting the disease early in its tracks [38]. In addition, we aimed to extend knowledge in this field by carrying out a comprehensive molecular assessment of MSC-EV-treated OACs, concentrating on their gene and microRNA expression profiles, to further elucidate the mechanisms of action driving the beneficial effects of MSC-EVs on chondrogenesis.

Our initial studies strengthened the growing evidence that MSC-EV treatment of OACs enhances their viability and ability to proliferate and migrate, as assessed by scratch and migration assays. Although most resident chondrocytes in healthy cartilage do not migrate, due to the high-tensile collagen network and pressurized matrix, biomechanical alterations in the diseased cartilage aid in their motility and migration to the damaged sites, which could be therapeutically exploited in cartilage regeneration [39]. In this respect, chondrocyte motility has been reported by several in vivo studies [40,41,42], suggesting that chondrocyte migration may be initiated by pre-existing OA damage. In relation to MSC-EVs, several groups have now reported enhanced chondrocyte migration upon MSC-EV treatment [6,10,43,44], in some cases in a dose-dependent manner [6,45]. This indicates the potential of MSC-EVs to facilitate chondrocyte migration during osteochondral repair, allowing for faster migration to the sites of damage. The mechanisms that mediate chondrocyte motility and migration in vitro and ex vivo have been assessed in several studies [46,47]. Serum has a chemotactic effect on OACs, which may be attributed to PDGF; however, additional factors aiding OAC migration such as IGF1 and MMP9 may play an important role [47,48]. While neither MMP9 nor IGF1 were significantly differentially expressed in our MSC-EV-treated OACs after 24 h of co-culture, the levels of MMP9 showed a positive trend, and it would be interesting to assess the expression profiles of migration-related genes over a longer period of MSC-EV treatment.

In accordance with others, we observed that OAC + EV demonstrated altered cytokine and catabolic protein production. In the 2D monolayer, OAC + EV showed reduced IL-8 and IFN-γ expression, and increased IL-13 production. Reduced IL-8 expression indicates a potential mechanism for MSC-EVs’ beneficial activity, by mitigating IL-8s osteoarthritic effects [49] where it has been shown to increase MMP-13, enhance NK-kB phosphorylation [50], and stimulate chondrocyte hypertrophy and calcification of the matrix [51]. Accordingly, IL-8 levels are higher in OA chondrocytes [52], synovial fluid [53], and serum [54] compared to healthy controls, further demonstrating a potential beneficial effect of MSC-EVs by reducing IL-8 expression in OACs, as observed in the current study. IFN-γ has been implicated in OA pathogenesis where it propagates inflammatory and degenerative events in chondrocytes by increasing inflammatory mediators (TNF-α, IL-6) and matrix-degrading enzymes (MMP-13) [55]. Our study suggests a pro-chondrogenic, anti-catabolic environment induced by MSC-EVs, by reducing the IFN-γ production levels of OACs. Regarding the anti-inflammatory role of IL-13, contradictory results have been reported dependent on the model employed. In an adjuvant-induced rat model of arthritis, IL-13 therapy reduced inflammation, vascularization, and bony destruction [56], while in an immune-complex-mediated arthritis mouse model, IL-13 overexpression diminished both chondrocyte death and MMP-mediated cartilage destruction, even though joint inflammation was enhanced [57]. In the present study, we observed decreased MMP13 gene and protein expression, along with IL-13 downregulation, in accordance with others [15,44,58,59], further suggesting a potential mechanism driving the beneficial effect of MSC-EVs.

To further assess the chondrogenic properties of MSC-EV-treated OACs, we adopted a 3D spheroid co-culture model. This more closely mimics the matrix-embedded in vivo environment and protects chondrocytes from dedifferentiation, essential for long-term cultures, while also facilitating MSC-EV uptake by OACs via enhanced diffusion, as reported previously [23]. Under these conditions, OACs treated with MSC-EVs displayed a chondrogenic-promoting effect, as demonstrated by sGAG quantification and toluidine blue histological evaluation, alongside increased ACAN gene and protein expression. In addition to the pro-chondrogenic increase in ACAN and aggrecan, the increased ATP levels detected may signify that MSC-EVs restore bioenergetic homeostasis in OACs by reprogramming their metabolic state, which could improve their survival and facilitate regeneration. These results reflect previous studies conducted in monolayer [14,44] or animal in vivo studies [59,60], whereby MSC-EVs show promise in modulating chondrocyte behavior and inflammatory responses, emphasizing their potential in cartilage repair.

We next adapted the in vitro 3D OA model23 to include inflammatory cytokine IL-1β stimulation, to mimic the pathological features of OA. We observed that the addition of MSC-EVs following IL-1β stimulation led to a significant increase in ACAN and COL2A1 expression, together with a decrease in MMP13 and ADAMTS5. We also observed that aggrecan was more homogenously distributed in the presence of MSC-EVs, confirming the trend of the gene expression results. Overall, the presence of EVs helped to reverse the effect of IL-1β, reflecting the findings of previous monolayer studies [14,15,17,44,58,61] and confirming the chondrogenic MSC-EVs’ beneficial effect in a more biologically relevant 3D model.

To further explore the molecular mechanisms underlying the enhanced chondrogenic potential of MSC-EV-treated OACs, we performed global gene expression profiling of OACs and OAC + EV cultured in monolayer and identified different transcriptomic profiles between OAC + EV and the controls, as well as distinct clustering of the two groups in a PCA analysis. Pathway analysis showed that the differentially expressed genes mapped to key processes associated with collagen biosynthesis and ECM maintenance. Previous studies have indicated that MSC-EVs mediated their beneficial cartilage repair effect by reducing the inflammatory response, while stimulating ECM production, thus restoring and maintaining cartilage homeostasis [7]. Our data are in keeping with this model, and we additionally show that several of the genes mapping to these pathways are validated targets of differentially expressed microRNAs identified in our study, including CCND1, COL5A1, COL5A2, SMAD3, and FGFR1. Although transcriptomic studies of the effect of MSC-EVs on OACs are lacking, Wang et al. performed RNA-Seq analysis of MSC cells co-cultured with chondrocytes in both 2D and 3D models [62], and pathway analysis revealed processes relating to early chondrogenesis and increased ECM interactions, in agreement with our data, in an MSC-EV model.

While previous studies have profiled the microRNA expression of MSC-EVs or chondrocytes, data to explore the microRNA repertoire of MSC-EV-treated OACs are lacking. Thus, we also provide novel insights into the changes that occur in OACs at a microRNA level in response to MSV-EV therapy, and potentially contribute to their mechanistic beneficial actions. Our data demonstrated 24 differentially expressed microRNAs, of which 7 demonstrated increased expression compared to the controls and have been previously associated with chondrocyte function.

MiR-125b demonstrates cartilage protective effects in OA, by directly targeting the collagenase MMP13 [63], and ADAMTS-4 to inhibit ECM degradation [64,65], while acting as a negative regulator of inflammatory genes including MMP-13 via targeting the TRAF6/MAPKs/NK-B pathway [66]. Our data corroborate this model in both monolayer and 3D cultures, further suggesting that increased miR-125b expression in OAC + EV may be partly driving MSC-EVs’ beneficial effects. Accordingly, miR-125b expression is significantly lower in OA vs. normal chondrocytes [67], further suggesting its role in OA pathogenesis, which may be reversed by MSC-EVs by targeting proteolytic enzymes. MiR-19a has been shown to promote cell viability and the migration of chondrocytes by upregulating SOX9, via the NF-KB pathway [68], and to suppress the catabolic factors ADAMTS5 and NOS2 [69]. Expression of miR-193a-5p is downregulated in OA cartilage tissue and chondrocytes compared to normal controls [70]. Its overexpression can suppress inflammation, apoptosis, and production of ECM in chondrocytes via suppressed IL-6, IL-1B, TNF-a, IL-8, blocked apoptosis of chondrocytes, induced expression of ACAN and COL2A1, and reduced MMP-3, MMP-13, and ADAMTS-5 expression [70]. MiR-1180-3p is significantly elevated in healthy controls compared to osteoarthritic models, suggesting a chondro-protective effect [71]. BM-derived MSCs express high levels of miR-1180-3p, which is critical to their induction of cell proliferation and glycolysis in an ovarian cancer model, where miR-1180-3p can modulate Wnt signaling by targeting SFRP1 [72]. This suggests a role for miR-1180-3p in the fine metabolic balance of chondrocytes, directly influencing chondrocyte hypertrophy and ECM degradation. MiR-140-3p is significantly downregulated in the serum, articular cartilage, and synovial fluid of OA patients compared to controls [73,74], is expressed in developing cartilage [75], and miR-140-deficient mice show age-related OA lesions characterized by proteoglycan degradation and articular cartilage fibrosis. It has been shown to protect chondrocytes from LPS-induced injury via inhibition of the NK-KB pathway [76], as well as suppressing the progression of OA by directly targeting CXCR4 [77]. Thus, in addition to regulating the development and homeostasis of cartilage tissue, miR-140 may have additional functions in inflammatory environments, such as OA cartilage [75]. Overall, our results directly demonstrating elevated expression of these microRNAs in MSC-EV-treated OACs further support their cartilage-protective properties and suggest a potential mechanism of action for MSC-EVs by mediating anti-apoptotic and anti-inflammatory pathways while enhancing ECM maintenance, anabolic factors, cell viability, and migration. This is reflected in both the target analysis of differentially expressed microRNAs, and our IPA integration of microRNA and gene expression profiles, linking to genes directly implicated in cartilage maintenance and development of osteoarthritis [78,79,80].

While the microRNAs and genes identified in our study as differentially expressed following MSV-EV treatment of OACs are in keeping with their known chondro-protective functions, it is now well recognized that the potency of MSC-EVs demonstrates significant heterogeneity depending on the donor cell [81]. In the current study, we used a mixed pool of n = 3 MSC-EVs for transcriptomics profiling, to minimize this variation and provide data reflective of MSC-EV heterogeneity. It would be interesting to assess the effects of individual MSC-EV populations with proven potency on the microRNA profiles of OAC + EV, and how heterogenic MSC-EV populations affect the transcriptomics of OACs regarding both gene and microRNA signatures.

To date, a significant limitation of the data reporting the effect of MSC-EVs on chondrogenesis is the lack of homogeneity between studies, particularly within the co-culture models used. Reviewing recent original studies, the majority used in vitro cultures [6,10,14,15,17,43,44,82], while a small number solely focused on in vivo EV injection in rabbit and pig models [60,83]. Of the in vitro co-culture studies, there is common disparity in the use of monolayer [6,10,14,15,17,43,58,82,84], 3D models [7], and alternative methods such as soft pellet co-culture [59]. There is also inconsistency in the use of inflammatory cytokine stimulation within the model, with many using IL-1β [14,15,17,44,58,59,82,84], but others using alternatives such as TNF-α [7] or not stimulating the chondrocytes [6,10,43], as well as variation in the dosage of stimulant, commonly ranging from 1 to 10 ng/mL [15,17,44,58,59,82,84], but also including doses as high as 10 mg/mL [14]. Within the monolayer studies that incorporated inflammatory cytokines, the duration of stimulation was commonly 24 h [7,14,15,17,44,58,59], but shorter (18 h [82]) or longer (72 h [84]) incubations were also employed. Finally, although most co-cultures used non-EV-depleted medium with FBS [6,10,15,43,58,82], the use of EV-free human serum [17] or non-EV-depleted human serum albumin [7] has also been applied. However, and importantly, despite the heterogeneity in study design and culture conditions employed, there is overall consensus in the data regarding the observed positive effects of MSC-EVs on OA cartilage repair and regeneration.

In conclusion, our findings provide important new insights on the potential of MSC-EVs as a promising treatment option for early-stage OA. We demonstrate some of the underlying molecular mechanisms responsible for the therapeutic effects of MSC-EVs and have performed full transcriptomic analysis of MSC-EV-treated OACs, which may pave the way for more targeted and efficient treatments that can enhance patient outcomes and overall quality of life. These discoveries and the insights generated by our study regarding the need for more standardized experimental models could inform and shape future research efforts, leading to a greater understanding of the potential for innovative therapeutic strategies in the early stages of OA.

## Figures and Tables

**Table 1:** Comparison of identified genes with SkeletalVis datasets. Genes identified as differentially expressed in OAC + EV that correlate with >3 published skeletal transcriptomic datasets (identified using SkeletalVis).

Direction	Gene Ensemble	Gene Name	Accession	Description	PMID	Species	Tissue
Up	ENSG00000156966	B3GNT7	GSE97118	Differentiation of Ezh2-deficient immature mouse chondrocytes	29280310	Mouse	Cartilage
	ENSG00000155980	KIF5A	E-MTAB-6266_B	Human knee OA subgroups and non-OA cartilage	29273646	Human	Cartilage
			GSE45233	Human injured meniscus with age and chondrosis	24692131	Human	Meniscus
	ENSG00000277377	SERPINA1	GSE55457	RA, OA, and control synovial membrane	24690414	Human	Synovium
			E-MTAB-1123	Mineralizing osteoblast-specific androgen receptor knockout	22525354	Mouse	Bone
			GSE45233	Human injured meniscus with age and chondrosis	24692131	Human	Meniscus
			E-GEOD-1919	Human OA or RA synovium treated with drug regimes	20858714	Human	Synovium
			GSE55584	RA and OA synovial membrane	24690414	Human	Synovium
	ENSG00000224212	TAP1	GSE27492	Synovial fluid cells from a mouse model of autoantibody-mediated arthritis timecourse	20506316	Mouse	Synovium
			GSE55457	RA, OA, and control synovial membrane	24690414	Human	Synovium
			E-GEOD-1919	Human OA or RA synovium treated with drug regimes	20858714	Human	Synovium
			GSE112413	Constitutively active FGF8 signaling in embryonic skulls	29752281	Mouse	Embryo
			E-GEOD-19664	OA chondrocytes and MSCs during chondrogenic differentiation	20883804	Human	Cartilage
			E-GEOD-1919	Human OA or RA synovium treated with drug regimes	20858714	Human	Synovium
			GSE55584	RA and OA synovial membrane	24690414	Human	Synovium
	ENSG00000235715	PSMB8	GSE27492	Synovial fluid cells from a mouse model of autoantibody-mediated arthritis time course	20506316	Mouse	Synovium
			E-GEOD-1919	Human OA or RA synovium treated with drug regimes	20858714	Human	Synovium
Down	ENSG00000137821	LRRC49	E-GEOD-19664	Osteoarthritic chondrocytes and MSCs during chondrogenic differentiation	20883804	Human	Cartilage
	ENSG00000122507	BBS9	E-MTAB-6417	Periosteum and bone marrow from fractured bones	29472541	Mouse	Bone

**Table 2:** Network analysis of differentially expressed microRNAs. Network analysis of significantly differentially expressed genes and microRNAs between OAC + EV and OAC. MicroRNA: gene interactions were identified using the IPA microRNA target filter, based on relationship confidence restricted to experimentally validated with inverse expression pairing. IEF = Ingenuity Expert Findings. Red colour indicates down-regulation, green colour indicates up-regulation.

MicroRNA	FC	Gene	FC	Source	Relationship		Pathway
miR-98-5p	↓	CCND1	↑	IEF	↓	↑	Cyclins and cell cycle regulation, IL-8 signaling
miR-98-5p	↓	COL5A1	↑	EF	↓	↑	GP6 signaling
miR-98-5p	↓	PRRC2A	↑	TarBase	↓	↑	
miR-98-5p	↓	SCYL1	↑	TarBase	↓	↑	
miR-125b-5p	↑	OSBPL9	↓	IEF	↑	↓	
miR-140-5p	↓	SMAD3	↑	miRecords	↓	↑	Human embryonic stem cell pluripotency, osteoarthritis pathway, IL-2 expression
miR-15b-5p	↓	CCND1	↑	IEF	↓	↑	Cyclins and cell cycle regulation, IL-8 signaling
miR-15b-5p	↓	FGFR1	↑	IEF	↓	↑	Human embryonic stem cell pluripotency, IL-15 production, osteoarthritis pathway
miR-23b-3p	↓	SMAD3	↑	miRecords	↓	↑	Human embryonic Stem cell pluripotency, osteoarthritis pathway, IL-2 expression
miR-29b-3p	↓	COL5A1	↑	IEF	↓	↑	GP6 signaling
miR-30e-5p	↑	COL5A2	↓	IEF	↑	↓	GP6 signaling
miR-30e-5p	↑	NCL	↓	TarBase	↑	↓	
miR-30e-5p	↑	PLOD2	↓	IEF	↑	↓	
miR-30e-5p	↑	SLC38A1	↓	TarBase	↑	↓	Glutamate receptor signaling

## Data Availability

The raw data supporting the conclusions of this article will be made available by the authors on request.

## Supplementary Materials

The following supporting information can be downloaded at: https://www.mdpi.com/article/10.3390/bioengineering11040388/s1, Table S1: TaqMan Gene and MicroRNA Assay IDs.
