# Supplementary material for: Functional and Molecular Analysis of Human Osteoarthritic Chondrocytes Treated with Bone Marrow-Derived MSC-EVs
Source: Bioengineering (Basel). 2024 Apr 17;11(4):388. doi: 10.3390/bioengineering11040388 (PMC11047960; doi:10.3390/bioengineering11040388)
Supplement: Supplementary file 1 [file bioengineering-11-00388-s001.zip › bioengineering-2925912-supplementary.pdf]

Table S1. TaqMan Gene and MicroRNA Assay IDs. All Assays were supplied by ThermoFisher Scientific.

**Gene/MicroRNA**	**Assay ID**
MMP13	Hs00942584_m1
SOX9	Hs00165814_m1
ACAN	Hs00153936_m1
COL2A1	Hs00264051_m1
COL10A1	Hs00166657_m1
ADAMTS5	Hs01095518_m1
GAPDH	Hs02786624_g1
miR-145-5p	002278
miR-29b-3p	000413
miR-21-5p	000397
U6	001973
HY3	001214
